# Supplementary material for: Hippocampal time cell dynamics evolve with learning to reflect cognitive demands
Source: bioRxiv. 2025 May 21:2025.05.20.655191. Preprint. [Version 1] doi: 10.1101/2025.05.20.655191 (PMC12140007; doi:10.1101/2025.05.20.655191)
Supplement: 1 [file NIHPP2025.05.20.655191V1-supplement-1.pdf]

bioRxiv preprint doi: https://doi.org/10.1101/2025.05.20.655191; this version posted May 21, 2025. The copyright holder for this preprint (which was not certified by peer review) is the author/funder, who has granted bioRxiv a license to display the preprint in perpetuity. It is made available under aCC-BY 4.0 International license.

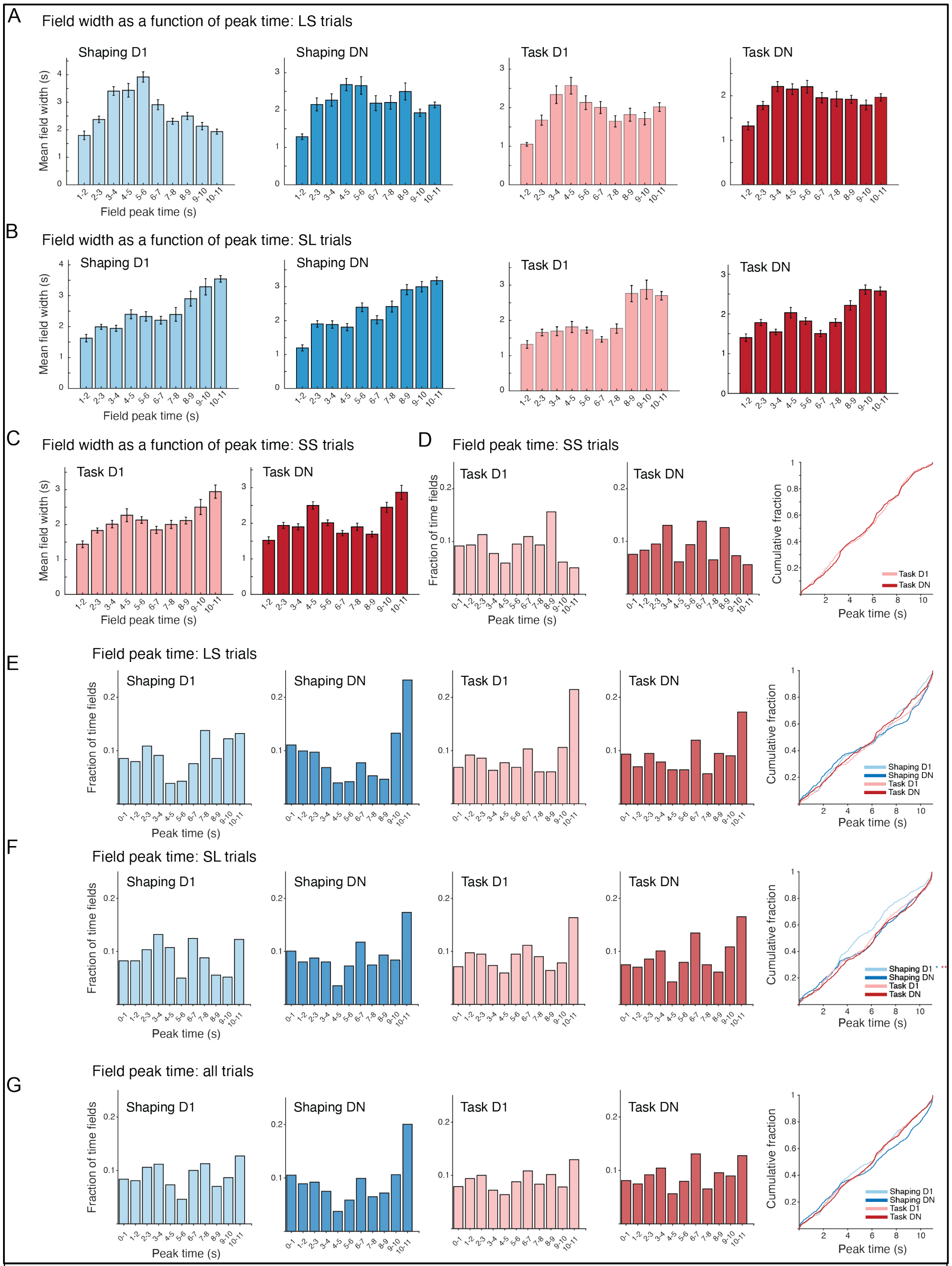

Figure S1. Additional analysis of time field peak and width. **A.** Time field width as a function of peak time for LS trials. Bars show average width of the time fields with a peak within each 1s bin. Bins span first odor onset (1s) to second odor offset. Histograms are shown for each session, and bars show mean ± SEM. **B.** Same analysis for SL trials. **C.** Same analysis for SS trials. **D.** Distribution of field peaks on SS trials. Left- histograms showing the fraction of time fields with a peak within each 1s bin of the trial, with first odor onset at 1s. Histograms are shown for each session. Far right- cumulative fraction plot comparing distributions across training phases (Kruskal-Wallis test with Dunn-Bonferroni post-hoc testing: *p < 0.05, **p < 0.01, ***p < 0.001 in all plots). **E.** Same analysis for LS trials. **F.** Same analysis for SL trials (p = 0.0032). **G**. Same analysis for all trials.

bioRxiv preprint doi: https://doi.org/10.1101/2025.05.20.655191; this version posted May 21, 2025. The copyright holder for this preprint (which was not certified by peer review) is the author/funder, who has granted bioRxiv a license to display the preprint in perpetuity. It is made available under aCC-BY 4.0 International license.

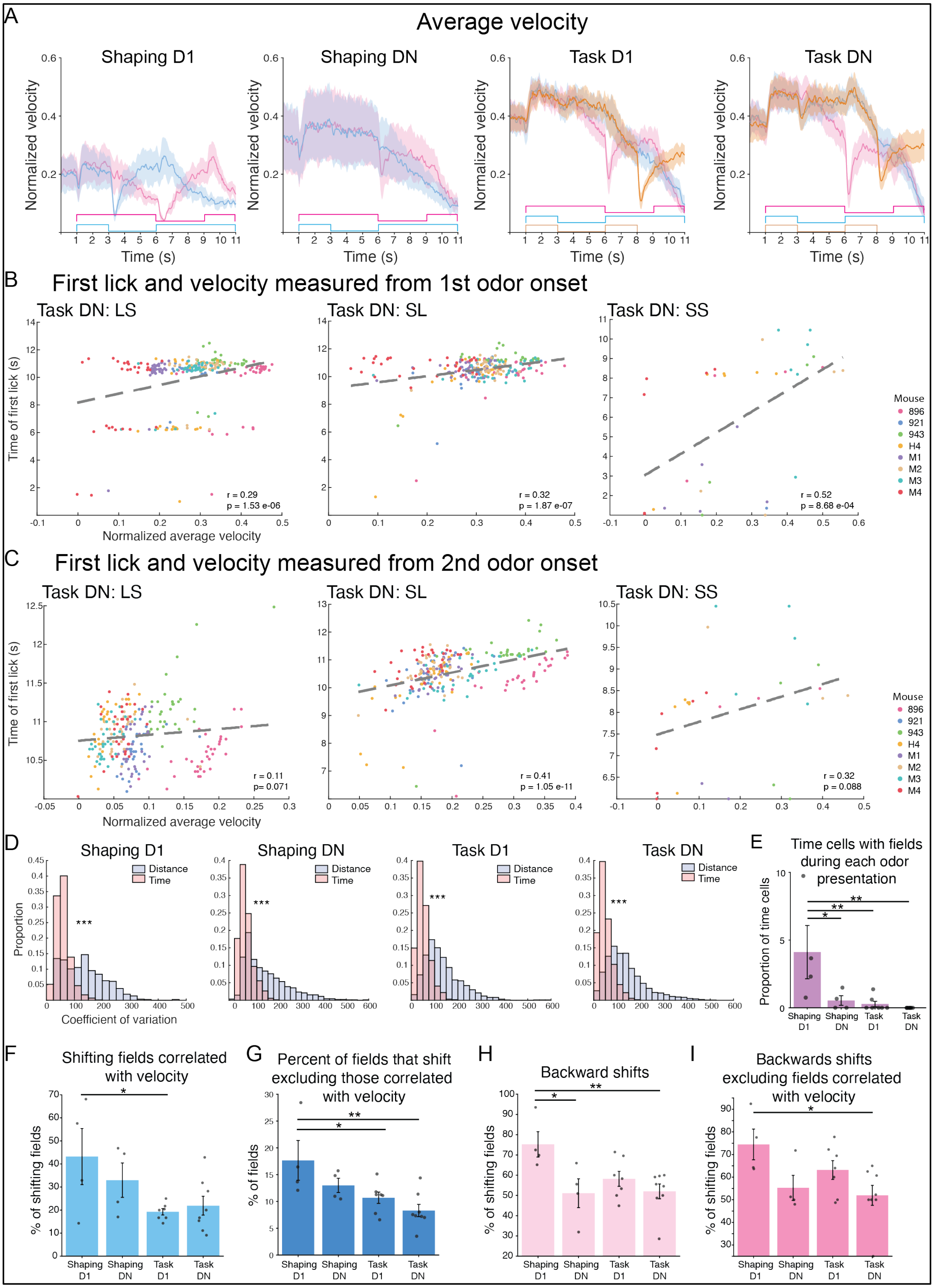

bioRxiv preprint doi: https://doi.org/10.1101/2025.05.20.655191; this version posted May 21, 2025. The copyright holder for this preprint (which was not certified by peer review) is the author/funder, who has granted bioRxiv a license to display the preprint in perpetuity. It is made available under aCC-BY 4.0 International license.

Figure S2. Time cell activity does simply reflect velocity or odor. **A.** Average velocity for each trial type and each session. Velocity was normalized by mouse to the maximum within the session. Lines depict average normalized velocity calculated across mice, and shading shows SEM (n = 4 Shaping D1, n = 4 Shaping DN, n = 7 Task D1, n = 8 Task DN). **B.** For each trial, the time of first lick and average velocity from first odor onset was measured. If mice track distance to solve the task, mice should lick earlier on trials with more running (higher velocity). However, across each trial type, the opposite relationship exists, where velocity is positive correlated with time of first lick ($p < 0.001$ for LS, SL, and SS trials). Each point represents a trial on Task DN, with dots colored to reflect mouse identity. **C.** Same as B, but with first lick and velocity measured from 2nd odor onset (p = 0.071, 1.05e-11, 0.088 for LS, SL, SS trials respectively; Pearson's correlation). **D.** Coefficient of variation measured as function of elapsed time or distance (from 1s before first odor onset). Distribution of values is shown for each session. In all sessions, the coefficient of variation is smaller when measured as a function of elapsed time ($p < 0.001$; paired t-test). **E.** Percent of time cells with a time field during each odor presentation in each context (p = 0.0013; linear mixed effects model with post hoc pairwise comparisons *$p < 0.05$, **$p < 0.01$). In all bar plots, dots show values for individual mice, with bars showing mean across mice ± SEM.**F.** Percent of shifting fields where field shift is correlated with velocity on a trial-by-trial basis (p = 0.022; linear mixed effects model) **G.** Percent of fields that shift, excluding all fields where shifts are correlated with velocity. Fewer fields shift with additional training (p = 0.0042; linear mixed effects model). **H.** Direction of field shift. The percent of shifts that are backwards (example in Figure 2D) decreases with training (p = 0.008; linear mixed effects model). **I.** Direction of field shift, excluding all fields where shift is correlated with velocity. The percent of backwards shifts decreases with training (p = 0.019; linear mixed effects model).

bioRxiv preprint doi: https://doi.org/10.1101/2025.05.20.655191; this version posted May 21, 2025. The copyright holder for this preprint (which was not certified by peer review) is the author/funder, who has granted bioRxiv a license to display the preprint in perpetuity. It is made available under aCC-BY 4.0 International license.

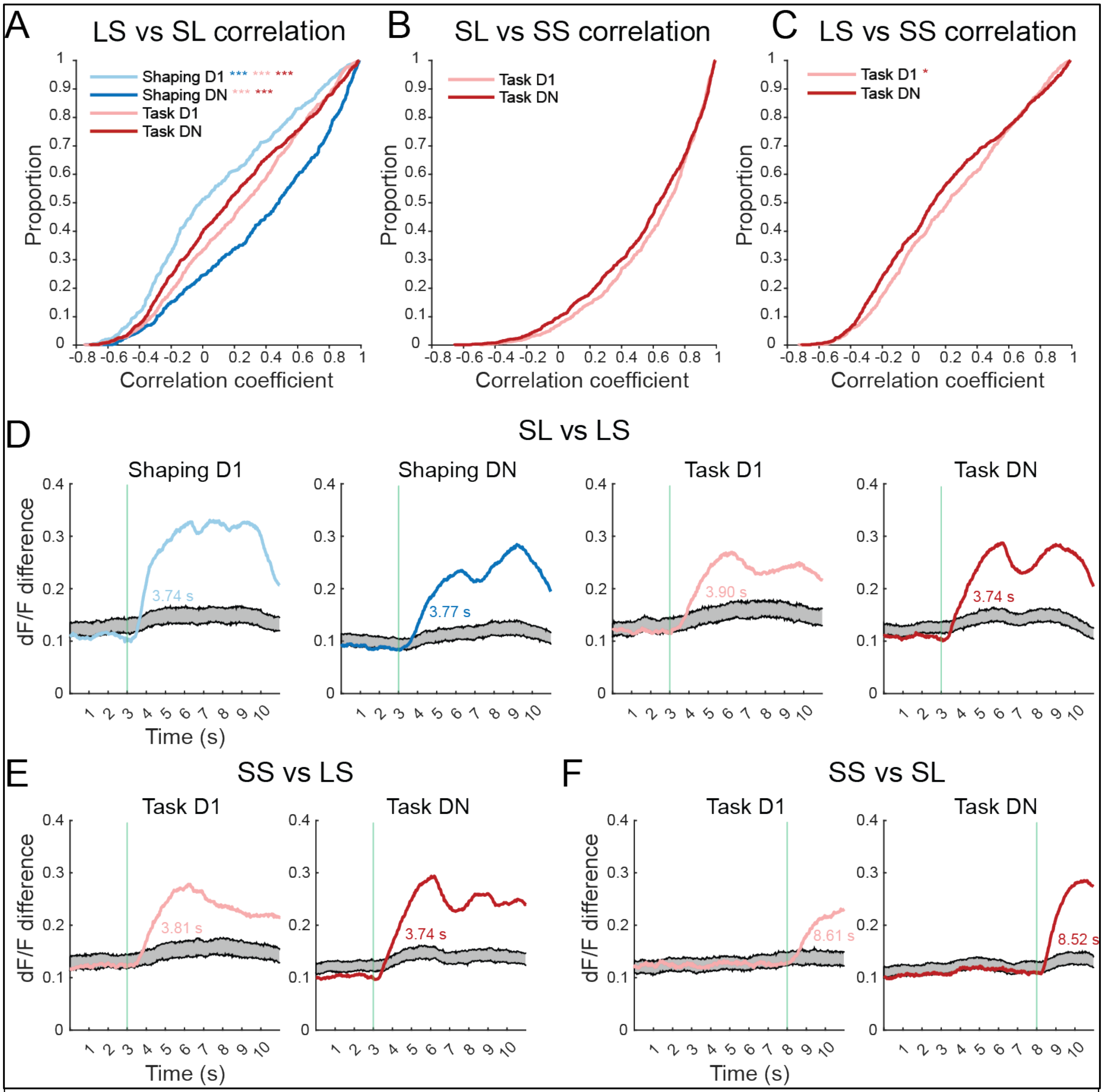

***Figure S3. Representations of context are distinct*.** **A.** Average correlation of time cell rate maps (Pearson's correlation) across LS and SL contexts, calculated for each session. Differences are significant across sessions (p = 7.53e-28; Kruskal-Wallis test with Dunn-Bonferroni post-hoc testing: *p < 0.05, **p < 0.01, ***p < 0.001 in all plots). **B.** Same as A but for SL and SS contexts (p = 0.054; Wilcoxon rank sum). **C.** Same as A but for LS and SS contexts (p = 0.012; Wilcoxon rank sum). **D.** Population vector difference in dF/F for time cells between SL and LS trials at each moment in time, shown for each session. Colored lines represent true data, and gray lines represent shuffled data distributions. Green bars demonstrate when stimuli diverge, and text indicates the time real data diverges from shuffled. **E.** Same as D but for SS and LS trials. **F.** Same as A but for SS and SL trials.

bioRxiv preprint doi: https://doi.org/10.1101/2025.05.20.655191; this version posted May 21, 2025. The copyright holder for this preprint (which was not certified by peer review) is the author/funder, who has granted bioRxiv a license to display the preprint in perpetuity. It is made available under aCC-BY 4.0 International license.

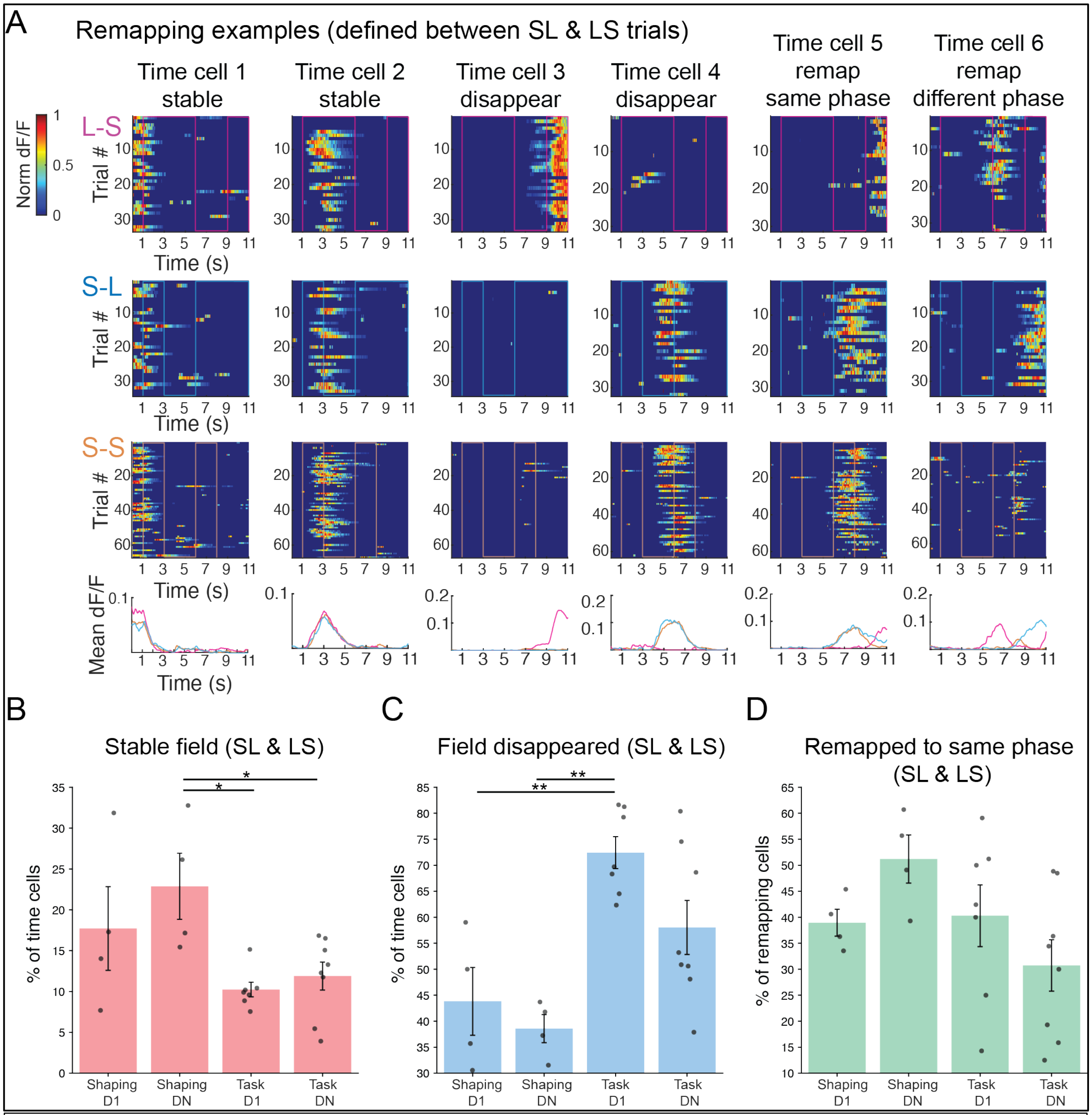

Figure S4. Time field remapping. **A.** Additional examples of time cells. Activity is shown for each trial, separated by trial type, with mean dF/F shown below. Time cell remapping was examined between LS (top) and SL (middle) contexts in this and subsequent plots. Some time fields are stable (cells 1 & 2), others disappear (cells 3 & 4) or remap (cells 5 &6) across contexts. Fields can remap to the same task phase (for instance, odor 2- cell 5), or different task phases (cell 6). **B.** Percent of time cells with a stable field decreases over training ($p = 0.0071$; linear mixed effects model with post hoc testing $^*p < 0.05$, $^{**}p < 0.01$). In all plots, data points represent percent of cells per mouse, with bars showing mean $\pm$ SEM across mice. **C**. Percent of time cells in which a field disappears between contexts increases with training ($p = 7.06e-04$; linear mixed effects model). **D.** The percent of remapping cells that remap to the same task phase does not significantly change across training ($p = 0.07$; linear mixed effects model).

bioRxiv preprint doi: https://doi.org/10.1101/2025.05.20.655191; this version posted May 21, 2025. The copyright holder for this preprint (which was not certified by peer review) is the author/funder, who has granted bioRxiv a license to display the preprint in perpetuity. It is made available under aCC-BY 4.0 International license.

522

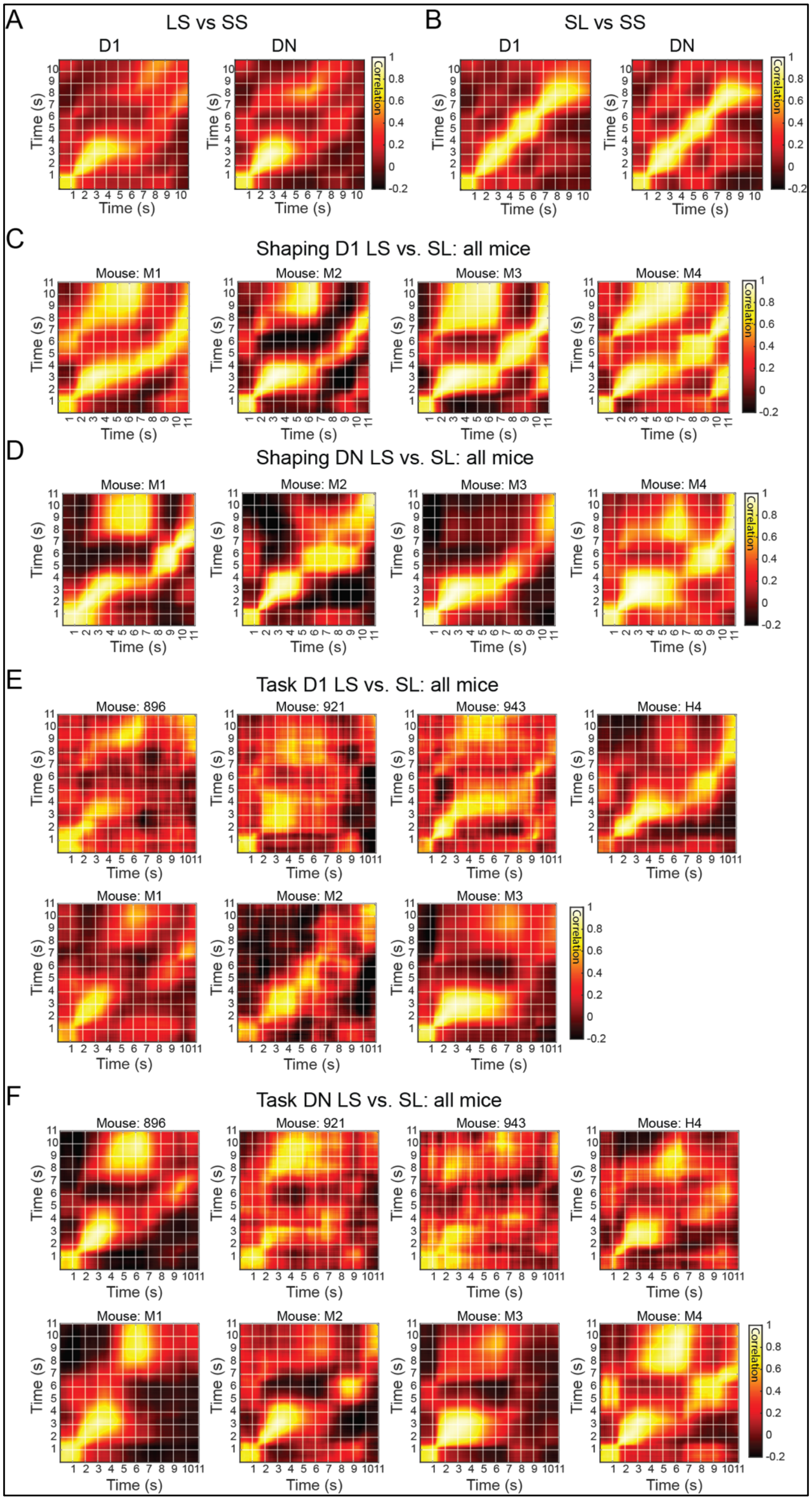

bioRxiv preprint doi: https://doi.org/10.1101/2025.05.20.655191; this version posted May 21, 2025. The copyright holder for this preprint (which was not certified by peer review) is the author/funder, who has granted bioRxiv a license to display the preprint in perpetuity. It is made available under aCC-BY 4.0 International license.

Figure S5. Additional cross-correlation matrixes.

A. Population vector cross correlation matrices, shown for LS & SS trial types across training phases (from left to right- Task D1, Task DN). **B.** Same as A but for SL & SS trial types. **C.** Population vector cross correlation matrices for each mouse, shown for LS & SL trial types on Shaping D1. **D.** Same as C but for Shaping DN. **E.** Same as C but for Task D1. **F.** Same as C but for Task DN.
